# Interrogating the Perceptions of Undergraduate Pharmacology Teaching on an MBBS Programme at a UK Medical School

**DOI:** 10.1002/prp2.70136

**Published:** 2025-07-25

**Authors:** Eleanor Renee Smith, Maximilian Paley, Raji Kaur Lalli, Maryam Malekigorji, John Broad

**Affiliations:** ^1^ Maidstone and Tunbridge Wells NHS Trust Maidstone Kent UK; ^2^ Faculty of Medicine and Dentistry Queen Mary University of London London UK; ^3^ Faculty of Life Sciences and Medicine King's College London London UK

## Abstract

Pharmacology education at medical schools in the UK aims to give newly qualified doctors the ability to apply foundational knowledge of pharmacology and to be able to prescribe drugs safely. This study aimed to assess a current pharmacology curriculum and understand the perspectives of both students and educators around pharmacology teaching. Employing a mixed‐methods approach, the research utilized documentation analysis, focus groups, semi‐structured interviews, and online questionnaires with students, educators and senior academic tutors. The analysis of the current curriculum revealed that 1069 drugs or drug classes were introduced to students in their first 2 years of study of drugs and drug classes. Students reported feeling overwhelmed with the number of drugs they were expected to learn. They suggested increasing contextual learning experiences and more practical prescribing experience. Students emphasized the need for greater visibility of pharmacology teaching. Students and educators identified challenges in integrating pharmacology effectively, which contributed to knowledge gaps. Disparities between students' perceptions of pharmacology education and educators' confidence in its delivery were found. These findings suggest the need to address the number of drugs introduced to students in their first 2 years of study. Recommendations include reducing the number of drugs or drug classes introduced to students, highlighting important drugs or classes, enhancing the visibility of pharmacology in the curriculum, and educating and supporting staff when preparing teaching sessions that involve pharmacology. These measures may address students' feelings of being overwhelmed by pharmacology, aligning with the aim of developing medical students into safe prescribers following graduation.

## Graphical abstract

Comparison of the perceptions of pharmacology teaching between phase 1 and phase 2/3 undergraduate MBBS students.

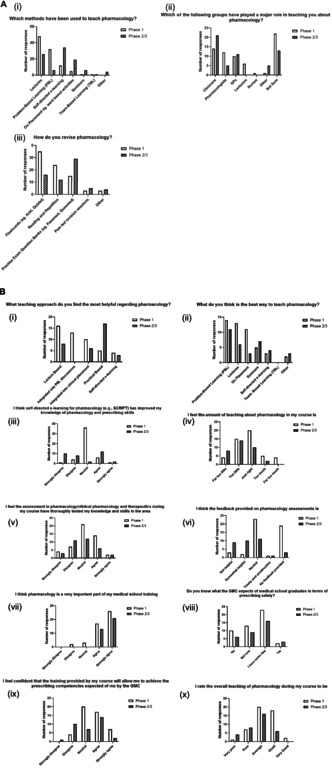

## 1 Background

Pharmacology education in the UK has undergone evolution since the 1960s, transitioning from traditional block teaching to an integrated approach following the General Medical Council's (GMC) [1] reforms. This reform aimed to spread pharmacology teaching across clinical years and various disciplines [2]. The way pharmacology teaching in UK medical schools is delivered has also changed, including the introduction of e‐learning modules, the incorporation of active teaching methods such as problem‐based learning (PBL) and team‐based learning (TBL), and the implementation of the Prescribing Safety Assessment (PSA) [3].

Despite these changes, studies highlight persistent concerns among medical students regarding their preparedness for practical prescribing [4, 5]. For example, Kennedy [6] found that only 36.4% of students felt adequately prepared for prescribing, echoing earlier studies that reported similarly low confidence levels [7]. Furthermore, the effectiveness of this integration has faced scrutiny [8]. While intended to reduce intellectual burden, integrating pharmacology into broader curricula may have inadvertently diluted its focus and reduced dedicated teaching time for pharmacology [9]. According to the GMC [10], newly qualified doctors must possess the skills to prescribe medicines safely (outcome 18) and be able to describe medications and medication actions (outcome 22e). An understanding of pharmacology is therefore foundational knowledge for doctors.

Recently, in our position as pharmacology educators, requests have been made from students and academic colleagues to assist students having difficulty with pharmacology and to produce drug lists for assessments. Due to the COVID‐19 pandemic, the 2020–21 academic year was taught entirely online. This presented a unique opportunity to undertake a comprehensive review of the learning resources provided to students to develop an understanding of the student experience of pharmacology teaching.

## 2 Study Aims

This study had three aims:

1. Review the first 2 years of course materials used to teach students on the Bachelor of Medicine, Bachelor of Surgery (MBBS) programme to understand the amount of pharmacology teaching that was delivered to the students.
2. Develop an understanding of students' perspectives of pharmacology education, focusing on course structure, teaching methods, and assessment and feedback mechanisms.
3. Develop an understanding of educators' views on pharmacology teaching in the course, with the aim of using these findings to inform future developments in early year curriculum design, ensuring better preparation for students as future prescribers.

The study also mainly focussed on the first 2 years of the MBBS programme as this aligned with the research team's lived experience, but the perspective of students from all years about this teaching was sought.

## 3 Methods

### 3.1 Ethical Considerations

Ethical approval was granted by the Institute of Health Sciences Education (IHSE) Research Ethics Committee at Queen Mary University of London (QMUL) for all aspects of this project. Approval from the head of year, acting in their capacity as the gatekeeper ensuring students are only enrolled in appropriate research studies, was also obtained at each step.

### 3.2 Context

The MBBS programme at QMUL is a 5‐year course, and is delivered in two campuses, one campus in London and one in Malta. During the first 2 years of the course (known as phase 1), students are predominantly taught through lectures, interactive sessions (anatomy, physiology, clinical skills, computer assisted learning and PBLs), and early clinical experiences in community settings. Students are taught in a spiral curriculum with modules that focus on body systems in health (year 1) and in disease (year 2). Student selected components were not included in our documentation analysis due to the high degree of variance in student experience. Students in phase 2 (years 3 and 4) have extended clinical placements and phase 3 (year 5) prepares students for practice.

Over the years of this study, approximately 350–550 students were enrolled in each year of the 5‐year course, across the London and Malta campuses. As the researchers were based at the London campus, only students and staff from this campus were enrolled in this study.

The primary data collection, including the documentation analysis, focus group/interview conduction, and questionnaire dissemination and analysis, was performed by medical students (E.R.S., M.P., R.K.L.) with an interest in medical education, all of whom had completed phase 1 at the time of the study. The students received both academic and simulated practical teachings on preparing for and facilitating focus groups and interviews as part of their intercalated Bachelor of Science (iBSc) in Medical Education. They were supported in the conceptualization and analysis of the data by non‐clinically qualified medical educators (M.M., J.B.) with an interest in pharmacology, who predominantly taught phase 1 students.

### 3.3 Curriculum Analysis

Our first aim was to understand the amount of pharmacology teaching that was contained in the course, and used mentions of drugs or drug classes as a proxy measurement of pharmacology teaching. To produce an estimation of the number of drugs or drug classes that were introduced to the students in phase 1, the content online was reviewed in its entirety. The lecture slide packs, PBL scenarios, practical session workbooks, and computer assisted learning packages were reviewed, and the names of the drugs or classes mentioned in the lecture slides were recorded. For the purpose of this project, both drug names and drug classes were included, as it is important for students to have an awareness of individual drugs as well as the properties of drug classes [8].

The frequency that these drugs or drug classes were mentioned across the phase 1 curriculum was calculated. The drugs or drug classes were also compared to the British National Formulary (BNF). The data set was then compared to the lists of “The Top 100 Drugs” that are prescribed in England [11, 12]. The purpose was to determine how many of these “essential” drugs were being taught in the phase 1 curriculum, as well as how frequently they are repeated. This comparison was based on the findings of De Vries et al. that the use of a core drug list and its associated prescribing information improved prescribing skills in medical students [13], ultimately lowering Foundation Year 1 (FY1) prescription errors and creating safer conditions for patients. Both the terms drugs and drug classes, are used by the authors of “The Top 100 Drugs” [12] when compiling their list. Therefore, more than 100 drugs were identified by this route. “The Top 100 Drugs” list actually contains 236 drugs and 110 drug classes‐ this is due in part to several drugs being part of one class, and the inclusion of important emergency drugs and commonly used IV fluid preparations in the list. “The Top 100 Drugs” and BNF lists used for analysis were ones that were used around the time of curriculum delivery. The analysis was also performed using the updated 2022 version of “The Top 100 Drugs” [14].

### 3.4 Student Focus Groups

To ascertain how the students felt about the pharmacology teaching in phase 1, focus group interviews of students studying in phase 1 (years 1 and 2) and phase 2 (years 3 and 4) were chosen as the research method. Interviews provide in‐depth qualitative data, offering rich, detailed insights into individual experiences and perceptions. Students were invited to take part by email invites, announcements at lectures, and through the Bart's and The London Student's Association. Semi‐structured questions for the focus group interviews were selected, as they had flexibility in how the interviewee was able to reply to questions whilst allowing the researcher to guide the interview as they saw fit [15]. The topic guide can be found in Table S1.

These semi‐structured interviews were conducted through the format of five focus groups of four participants per group (7 phase 1, 13 phase 2) lasting between 30 and 60 min. The same student investigator interviewed each group to allow for continuity between groups. The perspectives of phase 1 and 2 students were sought to provide both the perspective of students currently studying in phase 1 and a reflective view from those who have completed phase 1. These differing perspectives were sought to provide insight into whether students' views of the pharmacology teaching in phase 1 changed after they had experienced the teaching methods of phase 2. To provide the greatest possible flexibility for the participants, these focus groups were conducted online.

### 3.5 Senior Tutor Interviews

To ascertain the staff perception of how pharmacology teaching affected students, we decided to interview senior academic support tutors. Senior tutors are academics who have a good understanding of the entire MBBS curriculum and regularly meet students who are struggling with a particular aspect of the curriculum. Individual semi‐structured interviews were chosen as the method of data collection, as research by DeJonckheere and Vaughn [16] shows that they allow for the complex experiences to be heard, which could be considered in the conclusions of the research [17]. The topic guide can be found in Table S2. Of the four participants recruited, three worked as phase 1 and one worked as a phase 2 senior tutor.

All participants chose to conduct their interviews online. The interviews lasted between 19 and 39 min. Key topics discussed included students' perceptions of pharmacology teaching, the current benefits and challenges, the proportion of students who struggle, the reasons behind these struggles, and the available support for those students.

### 3.6 Student Survey

Given the limited sample size and the need to ensure that our findings from the interviews were representative and generalizable, it was decided to complement the qualitative data with surveys. The surveys were designed to capture a broader range of opinions from a larger population [18], allowing us to validate the themes identified during the interviews. This approach aimed to obtain a wider understanding of the current student perception of their own pharmacology education on this course.

An online questionnaire was designed to collect the opinions of medical students regarding pharmacology teaching across all 5 years of the MBBS programme. This questionnaire was adapted from one used in a study by Heaton et al. [7]. The questionnaire was further refined through discussions with pharmacology educators and focused on student learning experiences.

Students provided informed consent to take part in the survey, and participation was voluntary. The questionnaire included 14 mandatory questions and two optional open‐ended questions, which solicited specific opinions on pharmacology teaching and suggestions for potential improvements (see Figure S1). The open‐ended questions were intentionally made optional to prevent students from feeling compelled to provide responses merely to complete the survey. The questions were divided into four sections: year of study of the student completing the questionnaire, learning resources (multiple choice), opinions (Likert scale), and suggestions (free text response). For analysis, students were grouped into phase 1 (years 1 and 2 students) and phase 2/3 (years 3, 4, and 5 students) to allow comparison between students with early clinical exposure and students on extended clinical placements.

### 3.7 Educator Survey

A similar online survey was conducted for educators to gather their perspectives on pharmacology teaching and learning within the MBBS programme to assesses curriculum effectiveness and potential teaching enhancements. The educator questionnaire contained 12 mandatory questions and was aimed at educators employed at QMUL within the IHSE (the institute responsible for the design and delivery of the MBBS course, among others). The educator survey was also divided into three sections: demographics, opinions, and suggestions with same question format style (see Figure S2). The demographics section collected information about the educator's clinical or non‐clinical background and their years of experience in teaching pharmacology. Two open‐ended questions were included at the end to understand the perceived challenges in teaching pharmacology to medical students and to gather recommendations for improving their learning experience.

### 3.8 Data Analysis

Quantitative data were analyzed using Microsoft Excel and text responses were analyzed using the theoretical thematic analysis framework (coding and the development of themes) outlined by Braun and Clarke [19]. Transcription was aided by the transcription tool on Microsoft Teams, with manual checking of the transcription for accuracy by the student researchers. The analysis was performed manually for the focus groups and semi‐structured interviews by annotating the transcribed documents in Microsoft Word or Apple Pages.

The transcripts were coded and some of the themes were combined into one theme following application of Patton's [20] dual criteria of external heterogenicity and internal homogeneity. Some codes within themes were deemed to be too different and were therefore sorted into new themes.

After this, the themes were further defined and refined as described by Braun and Clarke [21] to identify their essence and determine what each theme could reveal about the data. During this process, subthemes emerged within certain overarching themes, such as the integrated curriculum, which included pharmacology's position as a subtheme.

## 4 Results

This section provides a summary of the data obtained from this mixed‐methods study of pharmacology education on the MBBS programme at QMUL. The curriculum analysis, student and staff focus groups, and surveys were completed between 2021 and 2024.

### 4.1 Curriculum Analysis

Students received 651 h of teaching delivered over 50 weeks, including 419 lectures and 46 PBL sessions in phase 1. Drugs or classes of drugs were mentioned in 169 lectures (40.33%) and 22 PBL sessions (47.83%). Only 15 drugs or classes of drugs (16.85%) were mentioned in physiology laboratory sessions, anatomy laboratory sessions, clinical skills sessions, histology sessions, and computer assisted learning sessions (in total 89 sessions).

A total of 1069 drugs or drug classes were mentioned across the phase 1 curriculum. Of those, 84 drugs or drug classes were mentioned four or more times, and 586 drugs or drug classes were mentioned once. Only seven drugs or drug classes in “The Top 100 Drugs” were not mentioned in phase 1 (Table 1). In the updated 2022 version this list remained the same [12, 14]. Drugs or drug classes contained in “The Top 100 Drugs” (2014) were mentioned in the phase 1 curriculum more frequently than those not contained in the top 100 drugs (3.44 mentions/drug in “The Top 100 Drugs” list, vs. 1.72 mentions/not listed drug). However, 146 of the drugs or drug classes mentioned in the phase 1 curriculum were not listed in the BNF at the date of access (27/05/22).

**TABLE 1:** Drugs/drug classes that are in the top 100 drugs list [12], but not taught in phase 1.

Drug name/class
Activated charcoal
Calcium carbonate, calcium gluconate, colecalciferol, alfacalcidol
Aqueous cream, liquid paraffin, emollients
Quinine sulphate, quinine
Hartmann's solution, compound sodium lactate
Potassium chloride

*Note:* 0.9%, 0.45% Sodium chloride solution.

### 4.2 Student Focus Groups

The theoretical thematic analysis of the focus group transcripts led to the development of three major themes as described below. The development of the themes can be seen in Table S3.

#### 4.2.1 The Integrated Curriculum

Many participants discussed the integrated curriculum, in which pharmacology teaching is interspersed with physiology, pathology, and other medical disciplines. Pharmacology was often viewed as a last‐minute addition to the curriculum. Several students also mentioned that pharmacology seemed less valued compared to other subjects, resulting in less time being allocated to it in the timetable (Table 2, Section A).

**TABLE 2:** Selected quotes from the focus groups.

Section A: Quotes evidencing student feelings on the integrated curriculum
“But they'll often mention pathology or a disease or something like that… And then they'll very briefly mention… Treatment and a lot of the time in that there'll be a drug name or a type of drug and they don't really go into it. They just sort of do it, oh, this is how it goes wrong. This is how you treat it. You don't need to know about that sort of thing…” (Iarq8) “SoI know a lot of the time you have lectures where they go through separate conditions like the pathophysiology of how they happen and then treatment is kind of thrown in as a little. Like an add‐on at the end” (8w35j) “There is no… there's no importance to pharmacology like you said, compared to Physiology, anatomy, clinical skills… They all have their own space that take up a weighted part of the curriculum, whereas with pharmacology. It's kind of swept under the; the rug” (Tsssb) “Yeah, it's quite hard to remember the pharmacology teaching cause I don't think it was very specific. I feel like each lecture had the drug sort of embedded within whatever topic they were talking about” (Fdxr9) “But I think if they give a back story… and then they're like, ‘So what drugs do you think you should she should be taking; taking into consideration and side effects things like that.’ I think that actually uses your brain to use its problem thinking skills and then it; and it feels okay: ‘This is why pharmacology is relevant obviously; like learning all of that is important’.” (0le2w) “And then you kind of when you go into placement, you see the drug charts. So you talk to patients, they tell you the drugs they're on…” (Jj3ak)

Section B: Quotes evidencing student feelings on the breadth of pharmacology teaching
“If they want a greater understanding of drugs in phase one, they need to very much rethink the curriculum in terms of mainly; in terms of volume.” (Cdfre) “I always felt like there was just a sudden list of drugs. And yeah, on one big slide of a presentation… in first year I remember being very overwhelmed with all the names of the drugs” (0le2w) “If I see a list of drugs: I don't wanna know. Especially if there's no context behind them” (Cdfre) “… maybe some of those [drugs] aren't the most important things that first and second years should be focusing on… Maybe it would be better to just focus on the; the ones that we actually should be retaining more.” (Ng6z7)

Section C: Quotes evidencing student feelings on what they value about pharmacology teaching
“Yeah, maybe some more awareness of what actually is used regularly in clinical practice ahead of going into third year would be good.” (Ng6z7) “Then a handful of other drugs that maybe had interesting mechanisms of actions were taught quite in depth even if they weren't particularly useful clinically…” (Cdfre) “At least for me, so far everything has been all my revision and even in pharmacology has been directed to: what do I need to know to get me to next year?” (Tsssb) “They don't emphasize the importance [of pharmacology] in first and second year, and then in 3rd year you don't know the names of any of the drugs the patients are on. And then you realize, oh, they actually are important.” (Jj3ak) “They didn't make it clear. What you would need and what you didn't.” (Jj3ak) “Whereas in phase one… I think that is it's still important to have this knowledge… but because it isn't really assessed, people don't necessarily learn it” (Cdfre) “These [pharmacology] are actually more important things that you need to learn because you actually have a whole exam on this [the PSA].” (Fdxr9) “I think that is it's still important to have this knowledge and understanding of pharmacology to underpin your knowledge when you go into phase two. But because it isn't really assessed, people don't necessarily learn it because even though it's interesting and it's one of the more interesting parts and people possibly want to learn it, there's this push and pull of what do I choose to learn” (Cdfre)

*Note:* These quotes were selected from the focus groups to represent student feelings on the themes of the integrated curriculum (A), the depth and breadth of pharmacology teaching (B) and what they value about pharmacology teaching (C). These themes were generated from subthemes identified in the focus groups transcripts. The alphanumeric participant identifier is found in brackets at the end of the quote.

A lack of specific pharmacology teaching was recalled as a complicating factor. In contrast, learning in context from case studies was mentioned as a positive aspect of the integrated curriculum. Some participants also reported that learning from patients, from drug charts and from prescribing guidelines received positive feedback from students.

#### 4.2.2 Breadth of Teaching

The vast number of drugs that the participants were exposed to in phase 1 was a recurring discussion topic. Many participants raised complaints regarding the use of lists of drugs with little context as a teaching method. The teaching of drugs deemed to be important was reported as a preference compared to being introduced to a large number of drugs (Table 2, Section B).

#### 4.2.3 What Do Students Value?

Participants raised concerns about the focus on drugs that are not clinically relevant, suggesting that time could be better spent on more commonly prescribed medications. Some also mentioned that an emphasis on less familiar drugs was not helpful. The concept of strategic learning was discussed, with participants expressing frustration over a lack of clarity and specificity in the teaching. Assessment, particularly the PSA, was identified as a key motivator for learning pharmacology (Table 2, Section C).

### 4.3 Senior Tutor Interviews

The theoretical thematic analysis of the focus group transcripts led to the development of four major themes (Table S4).

#### 4.3.1 Current Pharmacology Teaching

All participants mentioned shortcomings of the current pharmacology curriculum design, such as the volume of content students are expected to learn. Three tutors believed that the integrated design of the curriculum makes it harder to deliver pharmacology teaching, as there is a lack of clear guidance to staff on what should be taught and how. Two stated that this lack of guidance leads to inconsistencies in the aims and delivery of pharmacology across modules (Table 3, Section A).

**TABLE 3:** Selected quotes from the senior tutor interviews.

Section A: Quotes evidencing senior tutor feelings on current pharmacology teaching
“They understand principles of [pharmacology], I think it's more the vast variety that there is. There's so many drugs and there's so many modes of actions, so many off‐target affects, so many side effects… I think it's an issue of sheer volume.” (49cfb) “I'm just trying to think whether the list of lectures even includes anything that specifies, like, drug teaching as such. I can't think of any of my modules that I'm familiar with. It comes broadly into PBL, but it's not something that is a separate entity as such.” (wlwk7) “If there was more consistent, I guess, delivery and aims of pharmacology across different modules. Is it always going to be delivered by the same person, for example? Or in the same way?” (r0q4t) “I have heard there's some very good teaching before the prescribing exam towards the end of medical school. There's often a block that's really engaging and really useful.” (r0q4t) “I think that [AHPs'] education and training around pharmacology is significantly greater than medical students'… their depth of understanding of the principles, and their focus on prescribing, is a bit higher than we tend to do in medical school.” (bh45c)

Section B: Quotes evidencing senior tutor feelings on improving pharmacology teaching
“Maybe [pharmacology] is something that could be introduced more, and so students could then see the relationship? Maybe just touch on it in year one?” (wlwk7) “[Students] could have a bit of an introduction to, for example, drug classes, which would help them then have a framework in which to remember them… and maybe if someone explains why you get off‐target effects then it would make the effects easier to remember.” (49cfb)

Section C: Quotes evidencing senior tutor feelings on identifying struggling students
“I would say a good 30 to 40% of students struggle with [pharmacology], just by getting a general idea of feedback, mostly informal feedback, from students” (49cfb) “From conversations with students outside of my role as a senior tutor, so in my capacity as a lecturer, students definitely struggle with pharmacology… [but] it doesn't particularly come up in my senior tutor meetings.” (r0q4t) “If pharmacology was, say if there were pharmacology tutorials in small groups and it was a different sort of mode of teaching, I think students would separate it out… because they can usually highlight, say, “Oh I'm really struggling with the anatomy,” or “I'm really struggling with the histology.”” (r0q4t)

Section D: Quotes evidencing senior tutor feelings on the senior tutor programme
“I'll be honest with you, I never was trained into how to do the academic senior tutor role, except to say that we ought to act in a supportive role.” (49cfb) “There's no pharmacology lead that I'm aware of, so that isn't helpful… if a student came to me saying they were struggling with pharmacology I don't particularly know who to point them to, or who to get advice from. So, I do think that's a bit of a weakness.” (r0q4t) “To be honest, I don't know of anyone who specifically teaches pharmacology here.” (wlwk7)

*Note:* These quotes were selected from the semi‐structured interviews to represent senior tutor feelings on the themes of current pharmacology teaching (A), improving pharmacology teaching (B), identifying struggling students (C) the senior tutor programme as a whole (D). These themes were generated from subthemes identified in the interview transcripts. The alphanumeric participant identifier is found in brackets at the end of the quote.

Two participants were aware of the differences in pharmacology teaching across the years, specifically regarding PSA teaching. One participant discussed how allied health professionals (AHPs) often receive more dedicated classroom and workplace‐based pharmacology and prescribing teaching than medical students.

#### 4.3.2 Improving Pharmacology Teaching

Two participants suggested that pharmacology should be introduced to students earlier. It was also suggested that there should be more teaching on the core components of pharmacology to strengthen students' knowledge bases for when the content becomes more complex (Table 3, Section B). One participant argued that the current integrated nature of the pharmacology curriculum is preferable and mentioned that the medical school is trying to further integrate the curriculum.

#### 4.3.3 Identifying Struggling Students

Three participants stated that students frequently struggle with pharmacology but noted that many students had not discussed this during senior tutor meetings. Instead, this awareness came from conversations in their other capacities as educators. Participants also stated that, as phase 1 pharmacology teaching is entirely integrated, students can fail to identify it as an area of struggle in a way that is not true of anatomy and histology, both of which have dedicated teaching (Table 3, Section C).

#### 4.3.4 Student Support and Signposting

Two participants stated that they did not know who was responsible for overseeing and delivering the pharmacology curriculum. They reported that if a student presented with a pharmacology‐specific issue, they would not know how to refer them onwards to specialist colleagues (Table 3, Section D).

### 4.4 Student Survey

**FIGURE 1:**
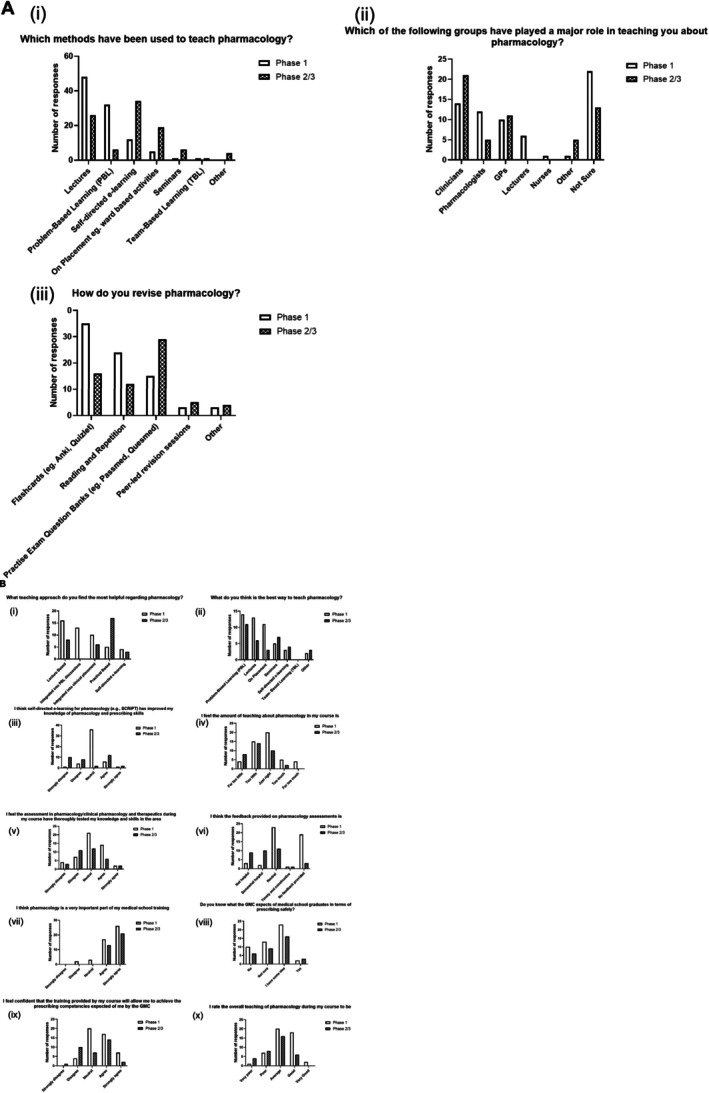
Comparison of the perceptions of pharmacology teaching between phase 1 and phase 2/3 students. Reponses of students to the category variable questions on the survey of student perceptions of pharmacology teaching on the MBBS programme at QMUL. Students were grouped by phases (12 students from year 1 and 36 from year 2 contributed to the 48 Phase 1 responses; 7 students from year 3, 21 from year 4 and 6 from year 5 students contributed to the 34 Phase 2/3 responses). In Section (A) students could select all options that applied, and in Section (B) students could choose one option only.

Eighty‐two medical students participated in the survey across the 5 years of the MBBS programme, an overall response rate of 4%. Phase 1 (years 1 and 2) had 48 respondents, phase 2 (years 3 and 4) had 28 respondents and phase 3 (year 5) had 6 respondents. Phases 2 and 3 (34 respondents) are grouped together for analytical purposes. The response to each survey item can be seen in Figure 1.

Students reported that pharmacology teaching mainly takes place in lectures (48 students) and PBL sessions (23 students) in phase 1, whereas more students reported learning pharmacology in self‐directed e‐learning sessions in phase 2 (34 students; Figure 1A (i)). 22 phase 1 students were unsure about who was responsible for pharmacology teaching. Phase 2 and 3 students more often identified clinicians (21 students) and GPs (11 students) as key educators (Figure 1A (ii)). Thirty‐five phase 1 students favored flashcards to revise, whereas phase 2/3 students predominately preferred using practice question banks (29 students; Figure 1A (iii)).

Phase 1 students found lectures (16 students) and PBL integration (13 students) most helpful for learning pharmacology (Figure 1B (i)). Phase 2/3 favored more practical approaches (17 students). Despite these differences, both groups agreed that PBLs were the most effective method for teaching pharmacology (Figure 1B (ii)).

In phase 1, most students (36) were neutral as to whether e‐learning improved their knowledge (Figure 1B (iii)). Fourteen phase 2/3 students agreed, and 18 phase 2/3 students disagreed that it improved their knowledge. Perceptions of teaching quantity differed between groups (Figure 1B (iv)): phase 1 students were divided, with 20 indicating “just right” and 19 feeling it was “too little” or “far too little.” In contrast, 22 phase 2/3 students felt the teaching was insufficient, while only 10 considered it adequate.

Most students were neutral when considering how thorough pharmacology knowledge and skills were tested in the course, but feedback on assessments was generally perceived as insufficient (Figure 1B (vi)).

All phase 2/3 students, along with 43 phase 1 students, agreed that pharmacology is an essential component in medical training (Figure 1B (vii)). However, awareness of the GMC's expectations for safe prescribing was low across both groups (Figure 1B (viii)). This uncertainty was mirrored in perception of preparedness: while 24 students in phase 1 students felt their medical school would equip them to meet GMC prescribing competencies, only 16 students in phase 2/3 felt the same, and 11 explicitly disagreed (Figure 1B (ix)). This suggests a lack of confidence in pharmacology training develops as students' progress.

Eighteen phase 1 students rated pharmacology teaching as good whereas students in phase 2/3 were more critical, with 12 rating it poor or very poor (Figure 1B (x)). Thematic analysis of open‐ended responses revealed the following areas of concern.

#### 4.4.1 Teaching Methods

Twelve phase 1 students and 20 phase 2/3 students completed free text comments. Five phase 1 and 10 phase 2/3 students expressed dissatisfaction with current pharmacology teaching methods, particularly the lack of explanations of drug mechanisms in lectures and the reliance on long, difficult‐to‐memorize drug lists. These concerns were echoed in student focus groups.

Seven phase 2/3 students were especially critical of the e‐ learning modules, describing them as “*extremely time‐consuming*,” “*overwhelming*,” and “*tedious*.” They called for reduced reliance on e‐learning, better accessibility for students with specific learning differences, and enhanced feedback mechanisms.

#### 4.4.2 Curriculum Integration

Two phase 1 students found pharmacology content to be poorly integrated, with one describing it as “randomly sprinkled into lectures.” Students in all phases suggested the inclusion of dedicated pharmacology resources, teaching days or standalone modules and earlier inclusion of the pharmacology content in the curriculum.

#### 4.4.3 Practical Experience

Seven phase 2/3 students would like more practical and interactive resources. One phase 1 student suggested “*it would be nice to receive a booklet with key drugs and relevant information*.”

### 4.5 Educator Survey

Six educators responded to the survey circulated to 20 academics within the IHSE, including clinical and medical sciences educators. Two had not taught pharmacology‐related content before, while the remaining had 1–10 years or more of experience. Three agreed that students are being adequately taught about safe prescribing, while three were neutral. There was no consensus over whether students feel confident using pharmacology skills (one agreed they do, three were neutral, one disagreed). All were neutral over self‐directed e‐learning, and 5/6 felt that the assessments thoroughly tested students' knowledge and skills. The responders thought that prescribers and pharmacologists should have a major role in teaching pharmacology. There was no consensus over the most effective teaching methods, but there was broad support (5/6 responders) for integrating pharmacology into each module rather than teaching it in dedicated blocks. Three suggested a slight increase in teaching, and three were neutral.

Challenges identified by educators included the vast number of drugs students must learn and the complexity of drug interactions, especially in polypharmacy. Educators suggested that students need more practical prescribing experiences and better communication skills for explaining prescription decisions to patients.

## 5 Discussion

This study used a mixed methods approach to understand the current state of undergraduate pharmacology education on the MBBS course at QMUL. A common theme raised by the curriculum analysis, focus groups, senior tutor interviews, and surveys includes concerns over the number of drugs introduced to students. Students expressed a desire to learn about fewer, more important drugs. Conflicting views were expressed by senior tutors on whether dedicated pharmacology teaching or further integration would help students become competent prescribers.

### 5.1 Curriculum Analysis

The document analysis revealed that a larger number of drugs and drug classes were introduced to students than anticipated by both the student and academic members of the research team. As expected, most of the drugs from “The Top 100 Drugs” list are introduced to students within the first 2 years of their studies, with notable exceptions such as emollients and various saline solutions (Table 1). Most drugs that foundation year doctors are expected to prescribe are introduced early in students' medical education. However, there are several drugs that do not form part of a junior doctor's regular prescription list. Notably, 13.6% of the drugs introduced to students were not available for prescription in the UK at the time of teaching. As it stands, this approach does not meet the recommendations of Ross and Maxwell [22], of “an initial focus in the early curriculum on understanding where and how drugs act.”

When preparing teaching material, educators should have the flexibility to introduce new and interesting therapeutic molecules to medical students. However, the cumulative effect of introducing drugs that are rarely prescribed by clinicians across 651 h of teaching is large and, as reported in the focus groups, affects students' perceptions of pharmacology education.

### 5.2 Focus Group

The student focus group participants reported that they enjoyed learning pharmacology in context, as opposed to simply reviewing lists of drugs on slides at the end of lectures. Learning in context is considered superior to other learning methods [23, 24, 25] and can be effectively implemented within an integrated curriculum [26]. Lerchenfeldt and Hall [27] argue that drug lists alone are insufficient for students to fully grasp the complex processes involved in prescribing the correct treatments. Participants also expressed a preference for learning about clinically relevant and commonly prescribed drugs. This further supports the suggestion of the development of a core pharmacopeia for students [13, 28, 29]. Focusing on frequently prescribed drugs aligns with the idea that familiarity with common medications reduces prescribing errors.

For pharmacology teaching to succeed within an integrated curriculum, it must be clearly identifiable, with well‐defined learning objectives [8]. Participants noted a lack of visibility for pharmacology within the integrated curriculum, which contributed to their perception that it was less important compared to other disciplines, echoing the concerns raised by Maxwell and Webb [30].

Another aspect of pharmacology education identified by participants was the overwhelming number of drugs they were expected to learn. This issue is not unique to the MBBS course investigated in this study. Maxwell [8] highlighted it as a common concern across medical schools, despite the GMC's recommendation [1] to reduce the factual burden in the medical curriculum. According to cognitive load theory [31], requiring students to memorize hundreds of drug names prevents them from developing the mental frameworks necessary to understand pharmacology as a whole. This redundant information imposes extraneous cognitive load, potentially reducing students' retention of the core drugs they will need to prescribe as foundation doctors. Additionally, some participants felt they were taught content that seemed unnecessary; when students perceive less face validity in their teaching, their engagement can decrease [6]. It is possible that the lack of clear responsibility for the oversight of the pharmacology curriculum within the integrated MBBS programme, as reported by the senior tutors, is a barrier to an improved curriculum.

Students were concerned about how they were assessed on the pharmacology teaching they received, and all groups discussed the impact of assessment on their learning. Assessments were not specifically mentioned in the topic guide (Table S1). Some explicitly stated they focused only on content they believed to be relevant for their exams, while others hinted at prioritizing topics they expected to be assessed. The large factual burden of the pharmacology curriculum encouraged some students to adopt strategic learning methods [32] to pass their assessments, leading to an incomplete, somewhat fragmented level of understanding of the curriculum [33].

### 5.3 Senior Tutors

The perceptions of the senior tutors aligned with the feelings of the student focus group participants in their concern over the overwhelming number of drugs introduced to students. The perceptions of senior tutors around the lack of formal identity of pharmacology as an educational theme, and a lack of individuals with a responsibility for the pharmacology curriculum, support Maxwell's [8] argument that the GMC's push for interdisciplinarity has negatively affected pharmacology education. The identification of individuals with a direct responsibility for pharmacology education across the programme (as recommended by Ross and Maxwell [22]) could help address these issues.

Several senior tutors also highlighted that many students struggle with pharmacology, which has been identified as one of the most challenging areas of medical education [34]. More students struggle with pharmacology than with other subjects overall [35]. Some tutors speculated that students may not identify pharmacology as an area of difficulty due to its integrated nature in both teaching and assessment. By contrast, subjects like anatomy and histology, which have dedicated teaching and assessments, are more easily recognized by students as areas of struggle.

The introduction of common elements between subject areas may help students relate new content to their prior knowledge [36]. Introducing integrated clinical pharmacology earlier in the curriculum through case based learning, for example [4], may help students develop stronger theoretical frameworks. These frameworks would serve as a semantic network, allowing students to assign meaning to new information. The stronger and more abundant the connections in this network, the easier it is for students to transfer knowledge across contexts [36]. According to this model, earlier clinical and more in‐depth pharmacology teaching should therefore benefit students.

While this approach may be challenging to implement within the integrated curriculum design recommended by the GMC [1], it is possible to include consistent, dedicated pharmacology teaching within the integrated curriculum. O'Shaughnessy et al. [37] note that 17% of British medical schools have resisted moving to a purely integrated curriculum. In our study, only one tutor advocated for further integration of pharmacology teaching, arguing that integrating content makes students more likely to apply that knowledge in clinical practice—an essential skill outlined by the GMC [10]. These conflicting views raise the question of whether a combination of integrated and discrete pharmacology teaching methods could be employed. The majority of students perceive problem‐, team‐, and case‐based learning sessions as preferable ways to learn pharmacology (reviewed in Fasinu and Wilborn [4]). The last full curriculum review of this MBBS programme was in 2012, and a curriculum review is currently in process. Future iterations of the MBBS curriculum could reduce the factual burden of memorizing drug names and prioritize the teaching of clinically relevant, regularly prescribed drugs within an integrated clinical context.

### 5.4 Surveys

The survey had a relatively low participation rate among students. This may be attributed to survey fatigue among medical students, a well‐documented issue in academic settings where frequent surveying can lead to declining response rates [38]. Students indicated that lectures, followed by self‐directed e‐learning and PBL, are the most common methods of learning pharmacology in the MBBS curriculum. However, students generally preferred PBL, aligning with studies highlighting its effectiveness in applied learning [39].

Students recognized clinicians as key contributors to pharmacology teaching but were often unclear about their specific roles, especially in the phase 1. This reflects a lack of a dedicated pharmacology course, which may enhance students' understanding of pharmacology [8]. Although e‐learning is used heavily in phase 2/3 for teaching safe prescribing skills, phase 2/3 students were divided about its effectiveness and some suggested reforms, contrasting with educators' more favorable views. Self‐directed learning, especially for complex topics like pharmacology, can be challenging if it lacks interactive elements [40].

Both students and educators stressed the importance of practical prescribing experiences for clinical readiness. The early clinical exposure that is included within the MBBS programme at QMUL provides an opportunity to learn pharmacology and prescribing in context. Students critiqued current drug lists, preferring explanations of drug mechanisms, which literature suggests could enhance engagement [41]. They also expressed dissatisfaction with pharmacology's integration into the broader curriculum, preferring dedicated teaching days or modules. This is in line with students' feedback obtained from the focus groups.

Students were neutral on whether pharmacology training met GMC competencies, while most educators believed the teaching was adequate. This disconnect could be resolved by improving communication around the expectations of the GMC around competencies, and for pharmacology gaining an identity within the integrated curriculum [42]. Co‐designing pharmacology teaching with students would offer a promising solution to the current challenges by fostering collaboration and ensuring that the curriculum better aligns with students' needs and learning styles [43]. By actively involving students in the design process, educators can gain valuable insights into what works well and identify gaps in content delivery, ultimately improving engagement and learning outcomes [44].

In summary, this study has identified some of the current challenges of pharmacology education for medical students at QMUL. These challenges are not unique to this course or this institution [4]. Future iterations of the MBBS curriculum could reduce the number of drugs introduced to students and emphasize clinically relevant drugs students will prescribe as foundation doctors. Utilizing the early clinical experience of students on this course to place drug treatments into context may emphasize the value of pharmacology to students.

### 5.5 Limitations

The research, conducted at a single medical school, has limited generalisability to other institutions. The pragmatic choice of focusing the document analysis on phase 1 teaching means that only the perceptions of the pharmacology curriculum as experienced by students in phase 2 and beyond were addressed in this study. Recruitment challenges for focus groups, with only four participants recruited per group, may have limited the breadth of perceptions that were obtained during the group sessions. Similarly, the small number of tutor interviews, particularly with only one tutor from phase 2, resulted in a limited and potentially non‐representative sample. We attempted to mitigate this limitation by using the survey. Self‐selection bias may have influenced participant views, and variations in curriculum delivery over the past 4 years could affect the findings. The small sample size of the senior tutor study further limits generalisability.

There is a risk of researcher bias due to personal experiences with the pharmacology curriculum, though this was partially mitigated by using topic guides. The involvement of student researchers, who had limited qualitative research experience, may have affected data quality, but their peer status might have encouraged more open discussions.

## 6 Conclusions

This study explored the perspectives of students and senior academic support tutors on pharmacology education on the MBBS programme at QMUL, as well as looking at the curriculum content related to pharmacology and a survey of educators and students. Shortcomings of the curriculum identified by participants include the exceptional number of drugs that are introduced to students (as supported by the documentation analysis), uncertainty over how to identify the important and relevant content, and how best to include pharmacology content as part of the integrated curriculum. Future research should focus on refining the pharmacology curriculum by investigating the long‐term impacts of curriculum changes on student outcomes, as well as evaluating the effectiveness of different teaching strategies, such as active learning and case‐based learning, in enhancing student understanding and retention of pharmacology content.

## Author Contributions

**Eleanor Renee Smith:** investigation, formal analysis, writing‐original draft, writing‐review and editing; **Maximilian Paley:** investigation, formal analysis, writing‐original draft, writing‐review and editing; **Raji Kaur Lalli:** investigation, formal analysis, writing‐original draft, writing‐review and editing; **Maryam Malekigorji:** conceptualization, methodology, visualization, data curation, writing‐original draft, writing‐review and editing; **John Broad:** conceptualization, methodology, visualization, data curation, writing‐original draft, writing‐review and editing, and project administration.

## Ethics Statement

This study was approved by the Institute of Health Sciences Education (ISHE) Research Ethics Committee at Queen Mary University of London (IPRCDec2021; QMERC22.117, IPREC221111.SMI, and IPREC231213.RAJ).

## Conflicts of Interest

The authors declare no conflicts of interest.

## Supporting information


Data S1.

## Data Availability

The data that support the findings of this study are available on request from the corresponding author. The data are not publicly available due to privacy or ethical restrictions.
