# Supplementary material for: Interrogating the Perceptions of Undergraduate Pharmacology Teaching on an MBBS Programme at a UK Medical School
Source: Pharmacol Res Perspect. 2025 Jul 25;13(4):e70136. doi: 10.1002/prp2.70136 (PMC12679945; doi:10.1002/prp2.70136)
Supplement: Supplementary file 1 — Data S1. [file PRP2-13-e70136-s001.docx]

**Supplementary Tables**

**Supplementary Table S1 - Topic guide for the semi-structured focus groups**

Table S1. This topic guide was used to shape the discussion in the focus group sessions for students. Additional follow up questions were permitted by the focus group facilitator depending on the responses.

**Supplementary Table S2** **- Topic guide for the Senior Tutor interviews**

Table S2. This topic guide was used to shape the discussion in the semi-structured interview sessions of senior tutors. Additional follow up questions were permitted by the interviewer depending on the responses.

**Supplementary Table S3** - **Thematic analysis of focus groups**

| **Codes** | **Sub-theme** | **Theme** |
| --- | --- | --- |
| Pharmacology is left to the end of the module or the lecture | Pharmacology’s position within the integrated curriculum | The integrated curriculum |
| Pharmacology is mentioned in passing in relation to a condition |  |  |
| Pharmacology has no specific teaching |  |  |
| Learning from case studies | Learning in context |  |
| Learning from patients |  |  |
| Learning from hospital drug charts |  |  |
| Learning from guidelines |  |  |
| Overwhelmed by the volume of information | Lists | Breadth |
| Lack of depth of teaching for most drugs |  |  |
| Dislike for drugs being presented in a list on a slide |  |  |
| Common or important drugs are poorly taught and understood | Clinically relevant | What do students value? |
| Drugs that are no longer commonplace in practice are taught in depth |  |  |
| Pharmacology seems unimportant in phase one | Do students care about pharmacology? |  |
| Pharmacology seems to be essential knowledge in phase two |  |  |
| The PSA as a major learning motivator | Assessment |  |
| Strategic learning of some drugs |  |  |
| If it is not assessed it’s a waste of learning time |  |  |
| Students want to be assessed on pharmacology in phase one |  |  |
| Lack of communication of what will and won’t be assessed |  |  |
| Unsure what is extra information and what is essential knowledge | Clarity |  |
| No emphasis of which drug to choose for which condition |  |  |

Table S3: The transcripts were recorded using Microsoft Teams and transcribed using the automated transcription software in Teams. The identification of codes, subthemes and themes was performed manually by highlighting codes in the transcript and constructing mind maps of the subthemes.

**Supplementary Table S4** - **Thematic analysis of Senior Tutor interviews**

| **Code** | **Sub-theme** | **Theme** |
| --- | --- | --- |
| Not had a student say they struggle with pharmacology | Students struggle to identify pharmacology knowledge deficits | Identifying struggling students |
| Students don’t identify pharmacology as a weakness |  |  |
| Students don’t see pharmacology as an independent topic |  |  |
| Students find pharmacology very difficult to conceptualise |  |  |
| Senior tutors receive a breakdown of students’ exam performance, but pharmacology is included in ‘core curriculum’ | A lack of academic performance data for senior tutors to identify struggling students |  |
| Aware of students’ struggles with pharmacology from their other academic roles, not from discussions as a senior tutor |  |  |
| Estimates, from other academic roles, that 30-40% of students struggle with pharmacology |  |  |
| Never discussed pharmacology in a senior tutor meeting | Pharmacology is not discussed during senior tutor meetings |  |
| Unaware of who oversees pharmacology teaching at BLSMD. | Issues with senior tutor training | The senior tutor programme |
| Would not know who to refer a student to if they had content-specific difficulties |  |  |
| Senior tutor training is fairly limited |  |  |
| Received no formal training on being a senior tutor |  |  |
| A lack of tutor-student continuity | Issues with the model of senior tutor support |  |
| Senior tutors are tied to an academic year not a cohort of students |  |  |
| Better student support existed previously (a large-scale academic support programme that followed students through) |  |  |

| Pharmacology is introduced too late | Challenges with the current pharmacology curriculum design | Current pharmacology  teaching |
| --- | --- | --- |
| More pharmacology in year one could increase student interest |  |  |
| Students struggle with the volume of pharmacology more than the content |  |  |
| Some modules are very drug-heavy compared to others |  |  |
| Pharmacology teaching is not consistent across modules |  |  |
| Never heard the current pharmacology curriculum design listed as a strength |  |  |
| Students may better understand pharmacology if it was a discrete module |  |  |
| Dedicated pharmacology SCRIPT e-learning modules begin in year 3 | Better pharmacology teaching is reserved for phases two and three |  |
| Better dedicated pharmacology teaching is reserved for later years |  |  |
| Allied healthcare professionals receive more in-depth pharmacology teaching | Medical students’ pharmacology teaching compared to allied health professionals’ |  |
| Allied healthcare professionals have more formal prescribing teaching |  |  |
| Allied healthcare professionals receive more regular pharmacology assessment and feedback |  |  |
| Introduce relevant pharmacology earlier | Changes to what is taught and when it is taught | Improving pharmacology  teaching |
| Adding pharmacology teaching to basic biochemistry to demonstrate relevance |  |  |
| Introduce drug class teaching |  |  |
| Teach the reasons for off target effects |  |  |
| Introduce small-group pharmacology teaching | Change the delivery of  pharmacology teaching |  |
| Introduce pharmacology specific lectures |  |  |
| Help students to develop the skills to become lifelong learners |  |  |
| Students appreciate the integrated curriculum design | Further integrate the  curriculum |  |
| Further integrate the pharmacology curriculum |  |  |

Table S4. The transcripts were recorded using Microsoft Teams and transcribed using the automated transcription software in Teams. The identification of codes, subthemes and themes was performed manually by highlighting codes in the transcript and constructing mind maps of the subthemes. BLSMD: Barts and the London School of Medicine and Dentistry; SCRIPT: Standardised Computerised Revalidation Instrument for Prescribing and Therapeutics.

**Supplementary figure S1: Medical Student Questionnaire about Pharmacology Teaching**

**Demographics:**

1. What year of Medical School are you in?
   1. 1
   2. 2
   3. 3
   4. 4
   5. 5

**Learning Resources:**

1. So far in your time at medical school which methods have been used to teach pharmacology? (Select all those that apply)
   1. Lectures
   2. Self-directed e-learning
   3. Problem-Based Learning (PBL)
   4. Team-Based Learning (TBL)
   5. Seminars
   6. On Placement, eg ward-based activities
   7. Other-please specify--------
2. Which of the following groups have played a major role in teaching you about pharmacology? (Select all those that apply)
   1. Clinicians
   2. GPs
   3. Nurses
   4. Pharmacologists
   5. Other- please specify--------------
   6. Not sure
3. How do you revise pharmacology? Select all that apply.
   1. Flashcards (eg. Anki, Quizlet)
   2. Reading and Repetition
   3. Peer-led revision sessions
   4. Practise Past exam questions
   5. Other- please specify--------
4. What teaching approach do you find the most helpful regarding pharmacology?
   1. Lecture based
   2. Practical based
   3. Integrated into clinical placement
   4. Integrated into PBL discussions
   5. Self-directed e-learning

**Opinions:**

1. I feel the amount of teaching about pharmacology in my course is:
   1. Far too much
   2. Too much
   3. Just right
   4. Too little
   5. Far too little
2. I think self-directed e-learning for pharmacology (e.g., SCRIPT) has improved my knowledge of pharmacology and prescribing skills.
   1. Strongly Agree
   2. Agree
   3. Neutral or I have not been provided self-directed e-learning for pharmacology yet.
   4. Disagree
   5. Strongly Disagree
3. I feel the assessment in pharmacology/clinical pharmacology and therapeutics during my course have thoroughly tested my knowledge and skills in the area.
   1. Strongly Agree
   2. Agree
   3. Neutral
   4. Disagree
   5. Strongly Disagree
4. I think the feedback provided on Pharmacology assessments is
   1. Timely and constructive
   2. Somewhat helpful
   3. Neutral
   4. Not helpful
   5. No feedback provided.
5. I think pharmacology is a very important part of my medical school training.
   1. Strongly Agree
   2. Agree
   3. Neutral
   4. Disagree
   5. Strongly Disagree
6. Do you know what the GMC expects of medical school graduates in terms of prescribing safely?
   1. Yes
   2. I have some idea.
   3. Not sure
   4. No
7. I feel confident that the training provided by my course will allow me to achieve the prescribing competencies expected of me by the GMC
   1. Strongly Agree
   2. Agree
   3. Neutral
   4. Disagree
   5. Strongly Disagree
8. I rate the overall teaching of pharmacology during my course to be
   1. Very Good
   2. Good
   3. Average
   4. Poor
   5. Very Poor

**Suggestions:**

1. What do you think is the best way to teach pharmacology?
   1. Lectures
   2. Seminars
   3. Problem-Based Learning (PBL)
   4. Team- Based Learning (TBL)
   5. Self-directed e-learning
   6. On Placement
   7. Other- please specify------------
2. Any other thoughts/opinions about the way pharmacology is taught in your course?
3. Any suggestions for pharmacology teaching in your course?

**Supplementary Figure S2: ISHE Educators’ Questionnaire**

**Demographics**

1. Which best describes your role?
   1. Medical Sciences Educator
   2. Clinical Educator
2. Number of years of experience in teaching pharmacology- related content:
   1. Less than 1 year
   2. 1-5 years
   3. 6-10 years
   4. More than 10 years
   5. My teaching does not include any pharmacology related content.

**Opinions**

1. I think medical students are being adequately taught about pharmacology and safe prescribing at Barts and The London Medical School.
   1. Strongly Agree
   2. Agree
   3. Neutral
   4. Disagree
   5. Strongly Disagree
2. I think our medical students feel confident using pharmacology knowledge and skills within in the clinical setting.
   1. Strongly Agree
   2. Agree
   3. Neutral
   4. Disagree
   5. Strongly Disagree
3. I think self-directed e-learning for pharmacology (e.g., SCRIPT) has improved medical students’ knowledge of pharmacology and prescribing skills.
   1. Strongly Agree
   2. Agree
   3. Neutral
   4. Disagree
   5. Strongly Disagree
4. I feel the assessments in pharmacology/clinical pharmacology and therapeutics thoroughly test medical students’ knowledge and skills in the area.
   1. Strongly Agree
   2. Agree
   3. Neutral
   4. Disagree
   5. Strongly Disagree

**Suggestions:**

1. Which of the following groups should have a major role in pharmacology teaching? (Select all those that apply)
   1. Clinicians
   2. GPs
   3. Nurses
   4. Pharmacologists
   5. Other- please specify--------------
2. Which teaching methods do you find most effective in teaching pharmacology to students? (Select all those that apply)
   1. Lectures
   2. Seminars
   3. Problem-based learning (PBL)
   4. Team-based Learning (TBL)
   5. Self-directed e-learning
   6. On Placement, e.g. ward-based activities
   7. Other- please specify------------
3. How do you think pharmacology teaching should be organised within the MBBS programme?
   1. Integrated throughout the content for each module.
   2. Separated into blocks where pharmacology is only taught on specific days/weeks in each module.
4. Would you change the amount of pharmacology teaching medical students are receiving at the moment?
   1. Significantly increase the training
   2. Slightly increase the training
   3. No change
   4. Slightly reduce the training
   5. Significantly reduce the training
5. What challenges do you see for students learning pharmacology and applying pharmacology related skills in clinical practice?
6. What improvements would you suggest that could be beneficial for students learning pharmacology?
